# The Biology of Monocytes and Dendritic Cells: Contribution to HIV Pathogenesis

**DOI:** 10.3390/v10020065

**Published:** 2018-02-06

**Authors:** Vanessa Sue Wacleche, Cécile L. Tremblay, Jean-Pierre Routy, Petronela Ancuta

**Affiliations:** 1 Faculté de Médecine, Département of Microbiologie, Infectiologie et Immunologie, and Centre de Recherche du CHUM, Université de Montréal, Montréal, QC H2X 0A9, Canada; vanessa.sue.wacleche@umontreal.ca (V.S.W.); c.tremblay@umontreal.ca (C.L.T.); 2 Chronic Viral Illness Service, Division of Hematology, McGill University Health Centre, Montréal, QC H3H 2R9, Canada; jean-pierre.routy@mcgill.ca

**Keywords:** monocytes, macrophages, dendritic cells, CD16, HIV, antiretroviral therapy

## Abstract

Myeloid cells such as monocytes, dendritic cells (DC) and macrophages (MΦ) are key components of the innate immune system contributing to the maintenance of tissue homeostasis and the development/resolution of immune responses to pathogens. Monocytes and DC, circulating in the blood or infiltrating various lymphoid and non-lymphoid tissues, are derived from distinct bone marrow precursors and are typically short lived. Conversely, recent studies revealed that subsets of tissue resident MΦ are long-lived as they originate from embryonic/fetal precursors that have the ability to self-renew during the life of an individual. Pathogens such as the human immunodeficiency virus type 1 (HIV-1) highjack the functions of myeloid cells for viral replication (e.g., MΦ) or distal dissemination and cell-to-cell transmission (e.g., DC). Although the long-term persistence of HIV reservoirs in CD4+ T-cells during viral suppressive antiretroviral therapy (ART) is well documented, the ability of myeloid cells to harbor replication competent viral reservoirs is still a matter of debate. This review summarizes the current knowledge on the biology of monocytes and DC during homeostasis and in the context of HIV-1 infection and highlights the importance of future studies on long-lived resident MΦ to HIV persistence in ART-treated patients.

## 1. Introduction

Cells of the innate immune system, such as myeloid cells (e.g., monocytes, macrophages (MΦ) and dendritic cells (DC)), contribute to the first line of defense against pathogens. Monocytes and DC originate from specific hematopoietic precursors in the bone marrow [1,2,3,4]. When homeostasis is perturbed, monocytes recruited at sites of inflammation give rise to distinct subsets of DC that will be able to rapidly stimulate effector T-lymphocytes. DC allow the early response against a particular pathogen to be translated into memory response with the activation of the adaptive immune system. HIV-1 exploits the features of monocytes and DC for its own replicative advantage. Although HIV-1 mainly infects CD4+ T lymphocytes, it also replicates in myeloid cells in various tissues [5,6]. Whether myeloid cells represent cellular reservoir for HIV remains highly controversial since no current studies have established viral persistence in myeloid cells of infected individuals after sustained control of viral replication using ART. Herein, we review the current knowledge on the biology of monocytes and DC as well as their contribution to HIV pathogenesis.

## 2. Monocytes Are Precursors for Macrophages (MΦ) and Dendritic Cells (DC)

Monocytes are bone marrow-derived mononuclear phagocyte cells that circulate in the blood for few hours/days before being recruited into tissues [7,8]. The expression of various chemokine receptors and cell adhesion molecules at their surface allows them to exit the bone marrow into the blood and to be subsequently recruited from the blood into tissues [9,10]. Monocytes are present in all vertebrates [11]. A similar population has been detected in fly haemolymph [12]. Monocytes represent approximately 10% and 4% of leukocytes in the human and mice peripheral blood, respectively, with a considerable pool located in the spleen and lungs, as well as homing into inflammatory sites in response to specific chemokines [11]. Monocytes belong to the innate arm of the immune system providing responses against viral, bacterial, fungal or parasitic infections [10,13]. Their functions include the killing of pathogens via phagocytosis, the production of reactive oxygen species (ROS), nitric oxide (NO), myeloperoxidase and inflammatory cytokines [13]. Under specific conditions, monocytes can stimulate or inhibit T-cell responses during cancer as well as infectious and autoimmune diseases. They are also involved in tissue repair and neovascularization [14].

Monocytes exhibit well-established developmental plasticity as they differentiate into MΦ, DC, or osteoclasts, depending on the inflammatory milieu [15,16]. Monocytes were originally considered the only precursors for MΦ and DC [9,11,17,18]. However, it is now well established that specific subsets of DC directly originate from bone marrow progenitors that are maintained independently of monocytes [2]. Also, most recent evidence demonstrated the existence of tissue resident MΦ of embryonic/fetal origin, with local precursors able to self-renew [3,19]; they include skin Langerhans cells [20]; liver Kupffer cells [21]; as well as peritoneal [22]; arterial [23] and brain MΦ [24]. The pool of resident MΦ coexists with the pool of monocyte-derived MΦ and the proportion between these two pools vary from homeostasis to inflammation and tissue injury [25]. For example, in the brain, microglia can be generated from monocytes under specific conditions when brain tissues are damaged [26]. Also, monocytes contribute to the replenishment of cardiac MΦ following local inflammation [27]. In contrast, in the intestine, the majority of MΦ and DC derive from blood monocytes that are continuously replenished [11]. These new insights in the ontogeny of myeloid cells may have profound implication for understanding disease pathogenesis and optimizing treatment.

Of particular importance, at steady state, monocytes traffic into tissues, capture antigens and return into the blood stream while retaining their “monocyte” phenotype [28,29]. This may explain the transcriptional and functional heterogeneity of monocyte subsets in the blood, subsets that represent distinct stages of differentiation [8,30,31,32].

## 3. Heterogeneity of Blood Monocytes

**Figure 1:**
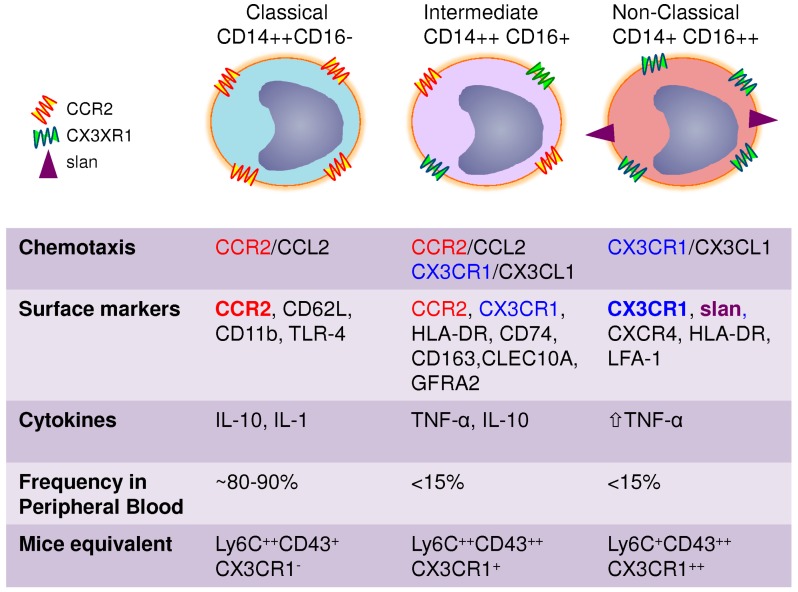
Human monocytes are classified into three subtypes based on the differential expression of CD14 and CD16: classical CD14++CD16− (also referred as CD14+ or CD14+CD16−), intermediate CD14++CD16+ (also referred as CD14+CD16+ or CD14+CD16int/low) and non-classical CD14+CD16++ (also referred as CD14LowCD16+ or CD14dimCD16+) monocytes.

Monocytes are the largest cells in the normal peripheral blood (14–20 µm diameter); they have particular morphology features that include an irregular cell shape, a nucleus with oval or kidney-like shape, cytoplasmic vesicles and a high nucleus to cytoplasm ratio (approximately 3:1) [33]. The original identification of monocytes was based on their morphology and cytochemistry, since monocytes are difficult to differentiate from blood DC, activated lymphocytes, or NK cells based on microscopic morphology only. The advances in flow cytometry allowed the proper identification of monocytes using size/granularity features and the expression of specific markers [34]. Flow cytometry also provided knowledge on the heterogeneity of the monocyte population. Human monocytes are now classified into three subtypes based on the differential expression of CD14 and CD16: classical CD14++CD16− (also referred as CD14+ or CD14+CD16−), intermediate CD14++CD16+ (also referred as CD14+CD16+ or CD14+CD16int/low) and non-classical CD14+CD16++ (also referred as CD14LowCD16+ or CD14dimCD16+) monocytes [11,33,35,36] (Figure 1). The main marker of human monocytes, CD14, is a glycoprotein and myelomonocytic differentiation antigen that functions as an accessory protein to toll-like receptor (TLR)-4 [37,38]. The CD16 (or the Fcγ receptor III) is a molecule of the Ig superfamily that is implicated in antibody-dependent T-cell cytotoxicity (ADCC) [37]. Originally, CD16 was reported to be expressed on NK cells and trigger target T-cell lysis [39]. In addition, CD16 is expressed on MΦ and neutrophils where it is involved in the uptake and clearance of antibody-opsonized pathogens [37]. The classical CD16− and intermediate/non-classical CD16+ monocytes represent ~90% and 10% of total monocytes, respectively [7,40]. Until the discovery of CD16+ monocytes in 1989 [34], monocytes were viewed as a homogeneous population [35]. Very recent studies elucidated the sequential differentiation and lifespan of human monocyte subsets and demonstrated that classical monocytes egress from the bone marrow into the blood stream where they give raise to intermediate monocytes that subsequently differentiate into non-classical monocytes [8].

For several years, the mechanisms regulating recruitment of CD16+ monocytes into the tissues where elusive; therefore, their functional role in inflammation and immunity was poorly understood. Indeed, it was reported that CD16− but not CD16+ monocytes expressed the MCP-1/CCL2 receptor CCR2 [41], known to be a major regulator of monocyte trafficking [42]. In 2003, studies by our group [43] and by Geissmann et al. [44] independently identified the fractalkine/CX3CL1 receptor CX3CR1 as a major regulator of CD16+ monocyte trafficking. Of note, CX3CR1 levels are higher on non-classical compared to intermediate monocytes, while classical monocytes expressed CCR2 and low/undetectable levels of CX3CR1 [43]. The CD16+CX3CR1+ monocytes migrate in response to soluble CX3CL1 but also attach under shear stress conditions to the CX3CL1 expressed on the surface of inflamed endothelial cells [43,45]. This interaction leads to the production of pro-inflammatory cytokines thus explaining the involvement of CD16+ monocytes in the pathogenesis of multiple diseases [46]. Subsequent genome-wide analysis of gene expression confirmed the preferential expression of CX3CR1 and CCR2 in CD16+ and CD16− monocytes, respectively [30]. All these findings opened the path for major advances in the field of monocyte biology and disease pathogenesis [17]. It is now well-established that monocytes subsets exhibit unique features. Classical monocytes express high levels of CCR2, CD62L, CD11b and TLR-4 with low levels of CX3CR1 [15,33]. In addition, a meta-analysis merging several transcriptional studies on monocyte subsets indicate that classical monocytes highly express low-density lipoprotein receptor (LDLR), scavenger receptor class B type-1 (SCARB1) and Stabilin-1 (STAB1) genes that may make the classical monocytes efficient scavenging cells [15,30]. Classical compared to intermediate and non-classical monocytes exhibit superior phagocytic and myeloperoxidase activities [33]. They also release heightened levels of superoxide. Non-classical monocytes are less granular, express high levels of the chemokine receptors CX3CR1 and CXCR4, the activation molecules HLA-DR and Lymphocyte function-associated antigen 1 (LFA-1), with low to undetectable levels of CCR2 and CD11b [35,41,43,47]. The differential expression of chemokine receptors suggests that classical and non-classical monocytes are recruited into distinct anatomical locations. In contrast to classical monocytes, non-classical monocytes produce very high amounts of TNF-α [48,49]. Non-classical monocytes appear to represent a more advanced stage of monocytes differentiation as they express transcripts typical for MΦ and DC [30]. Also, a transcription network meta-analysis suggested that non-classical monocytes are highly migratory cells since they exhibit elevated expression of genes involved in the cytoskeletal dynamic [15]. The expression of CD16 itself makes the non-classical monocytes a unique population. CD16 is known to activate the negative regulators of the TLR-MyD88-dependent pathway, rendering monocytes tolerant to sepsis [50].

Within the pool of CD16+ monocytes, the monoclonal antibodies M-DC8 bind to non-classical CD14+CD16++ monocytes and distinguish these cells from the intermediate CD14++CD16+ monocytes [51,52]. It was found that the M-DC8 antibodies (Abs) recognize the 6-sulfo LacNAc (SLAN) epitope [53], a carbohydrate modification of the P-selectin glycoprotein ligand 1 (PSGL-1) or CD162) [54,55]. The M-DC8+ population represents up to 40% of the non-classical monocytes. Of note, the highest production of TNF-α was detected in the M-DC8+ fraction within the non-classical monocytes following LPS stimulation [56]. Although non-classical CD14+CD16++ monocytes represent a small fraction of the total monocyte population in healthy individuals, this subset is significantly expanded in the blood of individuals with inflammatory conditions [33,35]. Excessive exercise and stress also leads to increased numbers of non-classical monocytes in the peripheral blood [57]. It is now well accepted that a developmental relationship exists between the classical and non-classical monocytes, including the fraction expressing M-DC8 [56,58,59,60].

Intermediate monocytes are a population that exhibit phenotypic and functional characteristics in between those of classical and non-classical monocytes [35,43]. Intermediate monocytes were not initially considered as a separate population and were originally allocated to classical or non-classical monocytes depending on the gating positioning in flow cytometry. Nevertheless, intermediate monocytes were later recognized as a distinct population from the two subsets previously found [35,40]. In fact, genome-wide transcriptional profiling demonstrated 90% of genes highly expressed in classical or non-classical monocytes were observed at intermediate levels in intermediate CD14++CD16+ monocytes [58]. Nevertheless, intermediate monocytes were shown to express higher levels of major histocompatibility complex (MHC)-class II including HLA-DR and transmembrane receptor CD74, the scavenger receptor CD163, the C-type lectin/C-type lectin-like domain member CLEC10A and Glial cell line-derived neurotrophic factor family receptor-α2 (GFRA2) [58,61,62,63]. Also, studies by Zawada et al. identified a higher expression of the angiopoietin receptor Tie2 [61]. The Tie2-expressing monocytes (TEM) were previously characterized as a subset of monocyte promoting angiogenesis [64,65]. The fact that intermediate CD14++CD16+ cells express the highest levels of Tie2 among the monocyte subsets indicates that this population may overlap with TEM [40].

Early studies by Frankenberg et al. demonstrated IL-10 mRNA expression in CD14++CD16− (classical monocytes) but not CD14+CD16+ (non-classical monocytes) in response to lipopolysaccharide (LPS) stimulation, while TNF-α, IL-1 and IL-6 transcripts were detected in both subsets [66]. Of note, CD14++CD16+ (intermediate monocytes) were not sorted/investigated in the later study. Most recent studies, demonstrated that intermediate monocytes produce the highest levels of IL-10 upon LPS or zymosan stimulation [67]. Despite some controversies related to the cytokines measured and monocyte subsets sorted, there is consensus on the pro-inflammatory features of non-classical and intermediate monocytes as opposed to their classical counterparts [35,68]. Other studies demonstrated that intermediate monocyte produce the highest level of IL-12 and IFN-γ in the context of antigenic presentation and have an increased ability to induce super antigen mediated T-cell proliferation [61]. This is in contrast to previous studies by Schakel et al. reporting that IL-12 production is associated with M-DC8+ monocytes [69], which are mainly non-classical monocytes [51].

Although some differences exist in the nomenclature of murine and human monocytes, explained by the availability of similar reagents for specific surface markers, the identification of monocyte subsets in mice similar to classical and non-classical monocytes allowed important mechanistic insights in monocyte biology with relevance for disease pathogenesis [2,17]. Murine monocytes are classified in two subsets: the Gr1+Ly6Chigh and Gr1−/Ly−6Clow. Of note, CX3CR1 is mainly expressed on Gr1−/Ly−6Clow, while CCR2 is highly expressed on Gr1+Ly6Chigh monocytes [17,44]. Thus, the classical and intermediate monocytes are closely related to Gr1+Ly6Chigh, whereas the non-classical monocytes are equivalent to the Gr1−/Ly−6Clow [13,33]. Based on the fact that only Gr1+Ly6ChighCCR2+ monocytes migrate in the peritoneal cavity in response to thioglycolate, while Gr1−/Ly−6ClowCX3CR1+ monocytes infiltrate various other tissues at homeostasis, Geissmann et al. proposed the term “inflammatory” for Gr1+Ly6ChighCCR2+ monocytes and “resident” for Gr1−/Ly−6ClowCX3CR1+ monocytes [70]. This classification is in part misleading because it implies that mice Gr1−Ly6CLowCX3CR1+ monocytes are not inflammatory; this contradicts a whole body of literature on the inflammatory potential of human CD16+ monocytes. Considering the fact that monocyte migration into the peritoneal cavity is dependent on CCR2, it is normal that CCR2+ but not CX3CR1+ monocytes migrate into this specific anatomic site. In fact, subsequent elegant studies by the group of Geissmann demonstrated that Gr1−/Ly−6Clow monocytes are the first line of defense against pathogens via the production of TNF-α and other pro-inflammatory cytokines as well as acting as “patrolling” cells on endothelial beds [47]. Therefore, the classification of monocyte subsets into “inflammatory” versus “resident” requires revision because all monocyte subsets can be either “inflammatory” depending on the specific chemotactic and activator stimuli they receive from specific tissues. Accumulating evidence indicates that the role of mouse Gr1−/Ly−6Clow and their human homologues, the non-classical CD14+CD16++ monocytes, is the surveillance of endothelial integrity. Indeed, intravital microscopy studies demonstrated the CX3CR1-mediated crawling of Gr1−/Ly−6Clow monocytes on the luminal side of the endothelium [47,71]. Also, Gr1−/Ly−6Clow cells were shown to be involved in the coordination of intraluminal stress response. These monocytes induce the recruitment of neutrophils that will promote endothelial necrosis and afterwards are involved in the clearance of the neutrophil-induced cellular debris. In contrast, the function of mouse Gr1+Ly6Chigh and human classical monocytes in the peripheral blood is poorly documented and is associated with their high phagocytic and scavenger activity [11]. Interestingly, it was recently demonstrated that Gr1+Ly6Chigh monocyte expression is under the control of circadian marker Bmal1 [72]. The same study also showed that these monocytes exit the bone-marrow in diurnal rhythmic waves. Such aspects require further investigations to establish a link between the alteration of circadian rhythms and the uncontrolled inflammatory features of monocytes during various inflammatory conditions.

## 4. DC Subsets and Associated Functions

DC represent a heterogeneous group of specialized antigen-sensing and antigen-presenting cells (APCs) that are essential for the induction and regulation of immune responses. The first DC were originally described in 1973 at a conference in Leiden by Ralph Steinman, an M.D. and Ph.D. candidate in the laboratory of Dr. Zanvil Cohn [73]. MΦ could only be distinguished from lymphocytes by their ability to adhere in culture dishes. Steinman et al. identified DC as cells able to extend or contract dendrites and named then DC as a reference to the Greek word dendron, which stands for tree. Steinman was awarded the Nobel Prize for this discovery in 2011. In the years following the original discovery, many types of DC were described in mice as well as in humans, including most recently the merocytic DC, a newly discovered DC subset with a unique ability to reverse T cell anergy and promote autoimmunity [74]. Several genetic comparisons succeeded in aligning mouse and human DC, supporting the hypothesis that human and mice have a similar organization of the DC system. In the peripheral blood, human DC are characterized as cells lacking the T-cell (CD3, CD4, CD8), the B cell (CD19, CD20) and the monocyte markers (CD14, CD16) but highly expressing HLA-DR and other DC lineage markers (e.g., CD1a, CD1c) [37].

**Figure 2:**
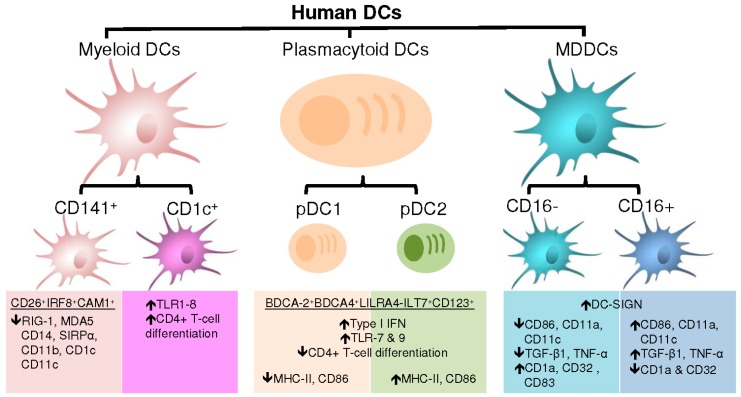
Human DC are divided into three major subsets: plasmacytoid DC (pDC), myeloid DC (mDC) and monocyte-derived DC (MDDC).

Similar to mice, human DC are divided into three major subsets: plasmacytoid DC (pDC), myeloid DC (mDC) and monocyte-derived DC (MDDC) (Figure 2).

### 4.1. Plasmacytoid Dendritic Cells

The pDC are distinct from mDC as they are long-lived specialized cells that massively produce type 1 interferons (IFNs) upon viral infection [9,75]. The transcription factor TCF4 plays a critical role in pDC lineage specification and effector functions [76]. In addition to inducing a wide range of responses affecting the function of multiple immune cells, type 1 IFNs produced by pDC directly block viral infections, resulting in pathogen clearance [75]. The pDC also can function as APCs that regulate T-cell responses but less potently than mDC. Human pDC can be detected by the expression of BDCA-2 and -4, leukocyte immunoglobulin-like receptor subfamily A member 4 (LILRA4-ILT7) and IL-3 receptor α-chain CD123 at the cell surface [77]. Under steady-state conditions, pDC have the morphology of an antibody-secreting plasma cell as indicated by their name [75,78]. As opposed to mDC, pDC have a narrow range of TLR expression. The pDC highly express TLR-7 and TLR-9 that detect viral and bacterial nucleic acids. Once activated via TLR-7 or TLR9, the pDC undergo changes in their morphology, with the acquisition of dendrites and up regulate the expression of MHC molecules and activation markers that will allow antigenic presentation to T-cells [79,80]. Indeed, pDC were reported to activate CD8+ T-cells [80]. However, pDC were shown to exhibit a low ability to activate CD4+ T-cells [75,81] possibly due to their limited ability for antigen uptake and presentation compared to mDC. The pDC can be divided in two subsets, pDC1 and pDC2 [75]. The pDC1 display an immature phenotype with low to undetectable expression of MHCII and activation markers, whereas pDC2 express highly MHCII and CD86. The pDC1 were reported to induce T-reg while pDC2 promote the differentiation of pro-inflammatory T-cells. Nevertheless, pDC1 and pDC2 are not stable lineages and can display plasticity between each other depending on local environment. Furthermore, ligation of CD123 on pDC has been found to result in the induction of iTreg producing IL-10. Interestingly, pDC were shown to promote Th17 differentiation [81]. Therefore, similar to mDC, pDC represent a distinct class of DC with a critical role in shaping the innate and adaptive immunity.

### 4.2. Myeloid Dendritic Cells

Often termed classical or conventional DC (cDC) in mice, mDC are specialized APCs with an extraordinary ability to sense and process antigens [9]. The mDC express CD4 but at lower levels compared to CD4+ T-cells [37]. Immature mDC have high phagocytic abilities, while mature mDC have an increased capacity to produce cytokines. Unlike mice mDC, human mDC can be detected in the peripheral blood [9]. The mDC are short-lived and constantly replaced by blood-derived precursors [82,83]. The mDC are highly migratory and regularly traffic from the periphery into T-cell and B-cell zones of lymphoid organs. At steady state or during infection, mDC regulate T-cell functions. The mDC are classified in two major populations based on the expression of CD141 (or blood DC antigen 3, BDCA-3) and CD1c (or BDCA-1): CD141+ DC (or cDC1) and CD1c+ DC (or cDC2) [35,84].

Human CD141+ mDC are found in peripheral tissues such as the lungs, skin, intestinal lamina propria and peripheral blood [84]. The CD141+ DC represent the mDC subsets with the highest capacity for cross-presentation [85,86]. Human CD141+ mDC are the homologues for mouse CD8α+CD103+ mDC that exclusively express the chemokine receptor XCR1 and the transcription factor IRF8. Both mice CD8α+CD103+ mDC and human CD141+ DC also express the cell adhesion molecule 1 (CAM1), activation receptor CLEC92A and TLR3. Also, human CD141+ DC conserved their function of being large producers of type III interferons. Although CD141 is also expressed in pDC, dermal CD14+ cells and CD1c+ DC, CD141+ DC are distinguished by their low to undetectable expression of cytosolic RNA or DNA sensors RIG-1 and MDA5 as well as surface molecules CD14, SIRPα, CD11b, CD1c and CD11c [84,87]. In addition, human CD141+ mDC express at high levels CD26 (or dipeptidyl peptidase 4) [84], a receptor for the enzyme adenosine deaminase and costimulatory molecules [88,89]. In fact, the combination of CD26, IRF8 and CAM1 are sufficient for the identification of CD141+ DC [84]. Anatomic locations can further define CD141+ DC. For example, intestinal CD141+ DC express the gut-homing marker CD103 (integrin αE). The CD141+ DC are known to express the co-stimulatory molecules CD40, CD80 and CD86 and upon maturation acquire the expression of CCR7 and CXCR3. The CD141+ DC migrate into the draining lymph nodes where they are specifically localized in paracortical regions [90].

Human CD1c+ DC are the most abundant mDC in the human peripheral blood [79]. The CD1c+ DC were also detected in lymphoid and non-lymphoid organs [84]. Human CD1c+ DC are the homologues of mouse CD11b+ DC [78,84]. Although all CD1c+ DC express considerable levels of CD1c, CD11c and SIRPα, the expression of several markers is dependent on their anatomic location. For instance, dermal CD1c+ DC express langerin, a marker of Langerhans cells [91], a type of antigen-presenting cell located in the skin. Also, dermal CD1c+ DC when compared to peripheral blood CD1c+ DC exclusively express CD1a and CD141 and down regulate the skin-homing epitope cutaneous lymphocyte antigens (CLA) [84]. In the same manner, lamina propria CD1c+ DC are distinguished from peripheral blood CD1c+ DC by their high expression of CD103 and CCR6 and lower expression of CX3CR1 as well as membrane glycoprotein CD64. Similar to CD141+ DC, CD1c+ DC express the co-stimulatory molecules CD40, CD80 and CD86 and acquire CCR7 expression upon maturation, a typical switch required for migration onto lymph nodes [92]. The CD1c+ DC distinguish from CD141+ DC by their expression of various TLR including TLR1-8. Whereas CD141+ DC were shown to induce Th1 and CD8+ T-cell differentiation [84,93], CD1c+ DC were found to generate not only Th1 but also Th2,Th17 and iTreg immune responses [84]. Indeed, mice cDC2 cells were reported as the primary inducers of Th2 and Th17 immune response following antigenic stimulation [94,95]. Mice cDC2 produce IL-23 during infection whether they are located in the lungs, skin, or within the intestine [94,96,97]. These particularities insure that each DC subset has a distinct role during host defense.

### 4.3. Monocyte-Derived Dendritic Cells

The discovery of monocytes as precursors of DC was made by Federica Sallusto and Antonio Lanzavecchia in 1994 [98] and further confirmed by others, including the group of Ralph Steinman [18,29,99,100]. Of note, MDDC exhibit similar transcriptomic features as CD1c+ DC [101]. In contrast to pDC and cDC, the development of MDDC is independent of Flt3L, a key regulator of DC commitment, consistent with the absence of Flt3 receptor on MDDC [33]. Both human and mice have MDDC [9,78,84]. Under steady state conditions, DC induction is independent of monocytes, while upon infection murine classical monocytes are recruited to the site of inflammation to be converted into “inflammatory” DC (inf-DC). The inf-DC have a large capacity to produce microbicidal compounds and appear to perform functions distinct from those of DC that are present under steady-state conditions [87]. Although inf-DC have been characterized phenotypically, distinguishing them from conventional DC and MΦ remains a challenge in vivo. It is thought that DC are shaped to induce tolerance during homeostasis thus preventing inflammatory responses from Th1 cells [102]. The inf-DC are thought to bypass this conditioning to become one of the first APCs capable of activating effector T-cells at the local site of infection [87]. Indeed, many research groups provided evidence that the immune system uses monocytes as precursors of DC for efficient antigenic presentation in the periphery during inflammation [103,104,105]. The MDDC are thus recognized as specialized APCs that potentially serve as an emergency plan in peripheral organs under hostile inflammatory settings. The Inf-DC are cleared from immune system once the inflammation is resolved [78]. The inf-DC produce large amounts of IL-23 upon specific stimulation leading to the differentiation of Th17 cells [106]. Following ligation of TLR-4, TLR-8, or nucleotide-binding oligomerization domain-containing (NOD) family members, inf-DC become a source of TNF and inducible nitric oxide (NO) synthase ((iNOS)-producing DC (Tip-DC)) with effective antimicrobial effector properties [78,87]. The MDDC can be generated in vitro from human and mouse monocyte precursors in the presence of IL-4 and GM-CSF (or CFS-2) [98,107]. The inf-DC are homologous to mouse and human MDDC generated in vitro [87]. Although not well described, inf-DC and Tip-DC were identified in humans with similar characteristics [108].

Human CD16− and CD16+ monocytes differentiate into DC in vitro [33]. Both MDDC subsets internalize soluble antigen and induce effector T-cells proliferation in similar manners [109]. In addition, human MDDC express at the cell surface CD11b, CD11c, MHC-II, Dendritic Cell-Specific Intercellular adhesion molecule-3-Grabbing Non-integrin (DC-SIGN), cell adhesion molecule CD24, SIRPα and macrophage markers CD107b and LAMP2 [33]. Indeed, DC-SIGN can distinguish MDDC from other subsets of DC [100]. Nevertheless, DC derived from CD16− and CD16+ monocytes are functionally distinct. Indeed, CD16+ compared to CD16− MDDC express higher levels of CD86, CD11a and CD11c as well as lower levels of CD1a and B cell co-receptors CD32 [109]. Also, CD16+ versus CD16− MDDC express higher levels of TGF-β1. The CD16+ MDDC were reported to preferentially differentiate into migratory DC upon transendothelial migration [110]. Furthermore, CD16+ MDDC induce the production of IL-4 following stimulation with TLR-2 ligand peptidoglycan, TLR-3 ligand poly-IC and TLR4 ligand LPS [111]. Conversely, CD16− MDDC primed with LPS express high mRNA levels of IL-12p40 mRNA and secrete high levels of IL-12 [109]. The CD16− MDDC also express high levels of DC maturation marker CD83 following ligation of TLR2, TLR3 and TLR4 [111], suggesting possible differences in the activation of signal transduction molecules between the two subsets of MDDC. Interestingly, a subset of DC distinct from CD1c+ DC and CD141+ DC referred to as Slan DC was identified in human peripheral blood [54]. These Slan DC are identified as CD1c−CD11c+CD16+CD14− cells that massively produce TNF-α, IL-12 and iNOS, as well as highly expressing TLR7 and TLR8 [112]. The group of Schäkel found that Slan DC produce high levels of IL-23, IL-1β and IL-6, which leads to the development of Th1/Th17 cells [113]. By being inducers of Th1/Th17 cells, slan DC are thought as pro-inflammatory DC that contribute to the pathogenesis of autoimmune diseases such as psoriasis. However, the identity of Slan DC as a distinct DC subset is still controversial since these cells are not detected in peripheral tissues and are genetically close to the monocyte lineage [114,115]. They are thought to be non-classical monocytes with DC properties [51,114]. In addition, MDDC were reported to possess the capacity of cross-presentation to naive T-cells and the ability to transfer peptides from MHC-I to lymphoid resident DC [116].

Peripheral blood monocytes represent an important pool of DC precursors that traffic to sites of infection. Monocytes are numerous in the blood and constitute an essential component in the induction of T-cell immunity. In fact, MDDC generated in vitro are the most common type of DC used in vaccine-based therapies [33,117,118].

## 5. Monocytes and HIV

During HIV/SIV infection, monocytes undergo changes in their frequency, phenotype and function. Indeed, monocytes expressing CD16 were first found to expand during AIDS, the late-stage of HIV infection [119,120,121]. The expansion of CD16+ monocytes was also reported during the chronic phase of infection [48,122]. This increase ranges from 30 to 50% in HIV-chronically infected and AIDS patients [48,119,122,123,124], as opposed to 5–10% observed in healthy individuals. This expansion may be due to high levels of monocyte/macrophage growth factor (M-CSF) observed in HIV-infected subjects [59], which was reported to induce monocytic maturation including the acquisition of CD16 expression [125,126]. The expansion of CD16+ monocytes was reported mainly in HIV-infected viremic individuals but not individuals receiving viral suppressive antiretroviral therapy (ART) [48,56,124,127,128]. The initial reports did not dissociate the intermediate from the non-classical monocytes. However, a most recent study reported that expansion of monocytes affected the non-classical population [129]. In contrast, Ancuta et al. observed the expansion of intermediate monocytes in a cohort of AIDS subjects with HIV-associated dementia (HAD) [124]. Of note, the expression of the HIV co-receptor CCR5 is the highest on intermediate monocytes [43]. Recently, emerging data confirmed the expansion of the intermediate monocytes in HIV-infected treatment-naive individuals and SIV-infected primates [56,130,131] and further documented their role during HIV pathogenesis [125,132,133]. Interestingly, the frequency of intermediate monocytes expressing CD163, a scavenger receptor, is increased in HIV-infected individuals with detectable viral loads but not in uninfected or in HIV-infected subjects with undetectable viremia [134]. The frequency of CD16+CD163+ monocytes is positively correlated with the viral load. Of note, the CMV status is associated with increased levels of soluble CD163 in the plasma of HIV-infected individuals treated with ART [135]. The frequency of intermediate monocytes positively correlated with levels of the inflammation marker IL-6, the monocyte activation marker soluble CD14 (sCD14) and the cardiovascular disease marker high-sensitivity C-reactive protein (hs-CRP) [136]. Therefore, this intermediate population may represent a biomarker for AIDS progression as well as non-AIDS related pathologies. The numbers of intermediate CD14+CD16++ cells are also increased during the acute and chronic phases of SIV infection [137]. Within the non-classical monocytes, the M-DC8+ fraction represent the most predominant population and contribute to the overproduction of TNF-α observed in HIV+ viremic individuals [56]. The expansion and infection of CD16+ monocytes can be decreased following Maraviroc intensification, a CCR5 inhibitor [138]. Furthermore, glucocorticoid treatment was shown to result in a decreased proportion of the CD16+ monocytes during SIV infection [130]. CD16+ monocytes expressing high levels of TLR4, particularly the non-classical monocytes, are major sources of IL-23 in response to commensal enteric bacteria such as E. coli during HIV infection [139]. Induction of IL-23 by CD16+ monocytes may contribute to systemic immune activation as it correlates with levels of immune activation marker CD27. Of note, the numbers of circulating classical monocytes were found unaltered during HIV infection by certain groups [48,56]. Nevertheless, Gama et al. identified the expansion of classical monocytes lacking the expression of CCR2 during HIV and SIV infection [140]. This enhancement was observed as early as seven days post primary SIV infection. CCR2low/neg classical monocytes harbored low levels of SIV-DNA compared to CD16+ monocytes but had defects in phagocytosis function and CCL2-mediated chemotaxis. In addition, CCR2low/neg classical monocytes suppressed CD8+ T-cells proliferation and IFN-γ release, indicating that these monocytes may postpone the development of the antiviral responses. The emergence of CCR2low/neg classical monocytes appears to be linked to viral replication, since ART initiation in infected primates and human individuals resulted in their decline [140]. However, Ancuta et al. reported a decreased frequency of classical monocytes in AIDS subjects with neurocognitive impairment but an increased expression of the HIV co-receptor CCR5 on these cells [124]. In this later report, HIV-DNA detection in FACS-sorted monocytes was a rare event, in contrast with the robust detection of the virus in CD4+ T cells isolated form the same in ART-treated individuals. Potential contaminations with CD4+ T-cells may explain controversies existent in the field regarding the presence of HIV-DNA in monocytes of ART treated individuals.

Studies by multiple groups including that of Suzanne Crowe demonstrated that CD16+ monocytes are more permissive to HIV infection and preferentially harbored HIV-DNA compared to CD14++CD16− monocytes even with successful ART [129,141,142]. This is consistent with the fact that CD16+ versus CD16− monocytes express higher levels of CCR5, which may facilitate HIV entry [124,142]. Of note, monocytes were previously reported to be resistant to HIV infection in vitro [143,144,145,146]. The HIV restriction in monocytes was described at the level of viral entry [145]. This HIV restriction was also localized at post-entry levels and explained by their limited ability to support HIV reverse transcription [147], due to low levels of dNTP available in monocytes for reverse transcription [144]. The function of SAMHD1 in cleaving dNTPs may explain the resistance of monocytes toward HIV infection [148,149]. Another HIV restriction mechanisms in monocytes is mediated by the beta-catenin/TCF4 pathway, which acts by restricting viral replication at transcriptional level [150,151]. The small frequency of CD16+ monocytes in the peripheral blood of uninfected individuals, together with the difficulties in working with such cell subsets, explains the limited knowledge in the field [126]. Permissiveness to HIV increases during monocyte differentiation [46,125,152,153]. Indeed, the induction of monocyte differentiation into MΦ leads to increased HIV permissiveness as opposed to freshly isolated monocytes [125,144]. Compared to classical CD16− monocytes, intermediate/CD16+ monocytes differentiating into MΦ were shown to promote HIV infection in CD4+ T-cells [154]. Genome-wide transcriptional profiling of CD16+ versus CD16− monocytes showed increased expression of syndecan-2 and fibronectin transcripts [125], two proteins previously reported to interact with HIV [155,156]. Syndecan-2 may promote viral entry as it acts as a trans attachment receptor for HIV [155]. Fibronectin was reported to interact with the HIV-gp120 protein thus facilitating virus-cell interaction [156].

Monocytes, particularly those expressing CD16, were reported to contribute to the initiation and persistence of neuroAIDS, a cognitive, motor and behavioral deficit pathology affecting the central nervous system (CNS) during HIV infection, also referred to as HIV-associated neurocognitive disorder (HAND) [126,157]. A correlation between the increased levels of circulating CD16+ monocytes expressing CD69 and HIV-related dementia was first reported in the late 1990’s [123,158]. Despite the success of ART, HAND affects 40% to 60% of HIV-infected individuals [159,160]. In fact, infection of monocytes correlates with HAND during successful ART [161]. The expansion of CD16+ monocytes persists even after one year of treatment [161]. Several lines of evidence support a model in which infected CD16+ monocytes migrate into the CNS where they differentiate into MΦ and act as cellular reservoirs for HIV [157]. Of note, levels of FKN/CX3CL1 are elevated in the brain of HIV-infected subsets and correlated with neurocognitive impairment [162]. Also, CD16+ monocyte recruitment into the brain is highly likely mediated via the FKN receptor CX3CR1 [43,70]. CD16+ mononuclear phagocytes expressing HIV antigens were observed in the perivascular space and within the brain parenchyma of HIV-infected subjects [163,164]; this supports the contribution of these monocytes to HIV seeding of the CNS. Myeloid cells are resistant to the HIV cytopathic effects, relative to CD4+ T-cells and monocyte-derived MΦ represent long-lived cells [165]. The infiltration of infected monocytes leads to chronic immune activation and inflammation within the CNS [157]. In addition, the transmigration of infected monocytes results in further infection of CNS associated cells including microglia, MΦ and, at low levels, astrocytes [126]. Although HIV does not productively infect neurons, they are affected by the consequences of HIV infection in the CNS. HIV-infected cells release viral proteins such as Tat and gp120, which results in the activation of surrounding cells including the MΦ, microglia and astrocytes [166]. The activated cells will in turn secrete cytokines such as IL-1β, IL-6 and TNF-α, as well as CCL2 and CXCL12 chemo-attractants recruiting more CD16+ monocytes in the CNS that will amplify neuro-inflammation [126,157]; neuro-toxic host factors such as arachidonic acid, quilolinic acid and NO will also be produced [167]. The presence of all these factors leads to deregulation of the blood brain barrier (BBB) permeability and astrocytic glutamate uptake; this will result in neuronal cell damage and death related to HAND. The transmigration of CD16+ monocytes is partly due to the increased expression of CCR2 [133]. The ligand of CCR2, CCL2 (or monocyte chemotactic protein 1 (MCP-1) is elevated in brain tissues of individuals with HAND [168]. Infected CD16+ monocytes appear to be highly sensitive to CCL2 in the induction of chemotaxis [133]. As mentioned earlier, the non-classical monocytes were previously reported to express low levels of CCR2 [35,43]. With the goal of amplifying CD16+ cells from the total monocytic population, Williams et al. established a protocol in which monocytes expressing CD14 and CD16 were generated following three days culture with M-CSF [125,133,157]. M-CSF functions as a factor differentiating monocytes into MΦ [169]. Cells generated following culture, which may be perceived as intermediate monocytes, express CCR2 and were found to transmigrate across the BBB in response to CCL2 in vitro [125,126,133]. In fact, the intermediate monocytes from HIV-infected individuals suffering with HAND expressed higher levels of CCR2 compared with HIV-infected subjects with unaltered cognition [132]. CCR2 levels were similarly high on classical or non-classical monocytes in the two groups of HIV-infected individuals. The enhancement of CCR2 on the intermediate monocytes was independent on ART, the severity of dementia, viremia and CD4+ T-cell counts/nadir. Furthermore, this increase of CCR2 observed in patients with HAND led to higher CCL2-mediated trafficking of intermediate monocytes into the BBB compared to counterpart monocytes from HIV-infected subjects with normal cognition. These results suggest that the intermediate monocytes play an important role in neurocognitive disorders and may be regarded as a biomarker for HAND [132]. This is consistent with findings by Ancuta et al. reporting preferential expansion of intermediate monocytes in AIDS subjects with dementia [124]. Another mechanism contributing to the intermediate monocytes entering the brain involved the expression at cell surfaces of junctional proteins interacting with the brain microvascular endothelial cells (BMVEC) of the BBB [126]. As the monocytes mature, they acquire increased expression of the junctional proteins such as junctional adhesion molecule-A (JAM-A), activated leukocyte cell adhesion molecule (ALCAM), CD99 and platelet endothelial cell adhesion molecule 1 (PECAM-1) [133]. Of note, HIV infection of intermediate monocytes results in an elevation of JAM-A and ALCAM, which, together with CCR2, were reported to mediate transmigration of intermediate monocytes across the BBB [133]. Consequently, JAM-A and ALCAM may be used as therapeutic target to inhibit the diapedesis of CD16+ monocytes across the BBB [170]. Other factors including osteopontin, an activation marker with chemotactic properties [171], may contribute to the maintenance of intermediate monocytes in the CNS. Osteopontin levels are elevated in HIV-infected individuals with dementia and also in intermediate monocytes [125,172]. Osteopontin was reported to decrease reverse transmigration across the BBB of CD16+ but not that of CD16− monocytes in vitro [173]; this mechanism potentially promotes selective retention of these CD16+ monocytes in the CNS during HIV infection. Targeting these surface molecules therapeutically may limit neuroinflammation during HIV infection.

The pro-inflammatory environment induced by HIV resembles that observed during the natural biological process of aging. Both HIV infection and aging affect the functions of the innate immune system [127]. Indeed, studies from the group of Suzanne Crowe showed that HIV infection leads to premature aged-related changes to monocytes [127]. The frequency, phenotype and functions of monocytes from young HIV-infected individuals are similar to those displayed by healthy elderly people [127]. In both groups, an expansion of CD16+ monocytes was observed. Also, monocytes from young HIV-infected individuals and elderly control individuals showed increased expression in CD11b and decreased levels of CD62L and M-CSF receptor CD115. Exposure of monocytes to TNF-α and LPS leads to decreased CD62L and enhanced CD11b expression at the cell surface, suggesting that the pro-inflammatory conditions induced during HIV-infection and aging may result in changes in the monocyte phenotype. The decrease in CD115 may be due to the high M-CSF levels observed in HIV-infected subjects leading to CD115 internalization and degradation. Furthermore, monocytes from elderly and young HIV-infected subjects have impairment in their phagocytic functions and displayed telomere shortening compared to cells from young uninfected controls [127]. Telomere shortening appears to reflect the increase turnover of monocytic precursors as monocytes do not typically undergo cell division. Indeed, monocyte turnover in SIV-infected primates is correlated to the innate immune activation marker soluble CD163 (sCD163) [174]. The increased CD16+ monocyte frequency, telomere shortening and changes in monocyte phenotype correlated with another innate immune activation marker, CXCL10 [127]; this indicates that CXCL10 may represent a biomarker for aging. The immune impairments and phenotypic changes in monocytes observed in HIV-infected individuals are not reversed by ART [122,124]. Most recent studies, revealed differences in myeloid cell responsiveness to TLR triggering between HIV-infected individuals with and without immunological restoration in response to ART [175]. Together, these lines of evidence reveal major alterations in the monocyte compartment during HIV/SIV infection. New questions emerge now concerning the impact of these alterations on the quality of the innate and adaptive immune responses since monocytes are well-established precursors for DC and MΦ.

## 6. Dendritic Cells and HIV

Primary HIV infection more frequently occurs via vaginal or rectal routes [176]. These mucosal tissues comprise a variety of DC subsets ready to encounter the virus. Due to their localization, DC play an important role in disseminating viral infection to CD4+ T-cells [177]. During early SIV infection, pDC are recruited to the site of infection following viral challenge [178]. This recruitment is mediated by CCL20 produced by endocervical epithelial cells. The pDC, in turn, secrete CCL3 and CCL4 leading to the trafficking of CCR5+CD4+ T-cells into the endocervix [178]. Furthermore, endocervical epithelial cells were demonstrated to secrete thymic stromal lymphopoietin (TSLP) in vitro, resulting in the activation of mDC that subsequently produce CCL17 and CCL22, further attracting naive CD4+ T-cells for expansion and infection [179]. Elevated levels of TSLP are associated with high viral replication in vaginal tissues of rhesus macaques during the first two weeks of SIV infection [179]. MDDC and mDC have also been found to capture HIV at the female reproductive tract [180]. Compared to CD4+ T-cells, DC are poorly infected [181]. Studies on early SIV infection demonstrated that resting CD4+ T-cells but not DC, represent the initial SIV-infected population in mucosal tissues [182,183]. Nevertheless, DC were found to sequester virus for several days before efficiently spreading the infection to CD4+ T-cells [184,185,186,187,188]. DC express high levels of the HIV-1 co-receptors CCR5 and CXCR4 and low levels of CD4 thus, allowing attachment and entry via the viral glycoprotein gp120 [189,190]. In addition to the expression of these key molecules for HIV entry, DC express other receptors capable of binding to the HIV-gp120 envelope protein. Indeed, myeloid dermal DC and MDDC express C-type lectin (CLR) receptor DC-SIGN as well as the mannose receptor [191,192,193,194]. Initially, studies by the group of Littman [195] demonstrated that DC-SIGN allowed HIV internalization into low pH sub-membrane vesicles thus preserving virion virulence [196]. The same group later published contradictory results that DC-SIGN was not essential for HIV *trans*-infection by DC [197]. The MDDC also express DC immunoreceptor (DCIR) that binds HIV-gp120 and contributes to HIV dissemination by DC to T-cells [198]. Other receptors such as syndecan-3 attach to gp120 and subsequently contribute to viral transmission [199]. Although pDC express the CLR BDCA2 and DEC-205, recognition and binding of HIV-gp120 is mediated by CD4 followed by the endocytosis of viral particles [200]. The HIV can also enter in DC independently of HIV-gp120. Interaction between glycosphingolipids in the virus lipid bilayer and an unknown receptor at the DC surface membrane were reported to occur [201,202,203]. Lipid content on DC membrane surface may affect envelope-independent HIV capture. Indeed, peroxisome proliferator-activated receptor (PPAR)γ and liver X receptor (LXR) prevent HIV uptake by triggering cholesterol efflux which diminishes the lipid content [204]. The maturation of both MDDC and mDC results in an increased HIV dissemination infection potential [205].

The DC play a dual role in HIV dissemination via infection in *cis* and/or *trans*. *Cis*-infection occurs when HIV infects DC as target cells, generating virions de novo [206]. This phase lasts 24 to 72 h after HIV exposure [176]. Indeed, low levels of productive HIV infection occurs in DC [207]. Nevertheless, DC excel in their ability to capture HIV and facilitate infection of CD4+ T-cells [185,193,208]. The transfer of virus from DC to CD4+ T-cells is referred to as HIV *trans*-infection. Transmission of viral particles from DC to CD4+ T-cells involves exosome secretion pathway or structures called virological/infectious synapse [209,210,211,212,213]. More precisely, the virological synapse is defined as an adhesive junction between two cells (one infected and one uninfected) involving the cytoskeleton and lipid rafts that allow transmission of a virus from a donor to a recipient T-cell [214,215]. The first documentation of the virological synapse was made in the context of human T-cell leukemia virus type 1 (HTLV-1) transmission between infected and uninfected T-cells [216]. Viral transmission by DC occurs when a DC loaded with HIV is in the proximity of a T-cell allowing the formation of a cell-contact in the context of antigen presentation [209]. The virological synapse between DC and T-cells is strengthened by molecules such as DC-SIGN, ICAM-1, LFA-1 and CD4 that are also involved in the formation of the immunological synapse [208,209,217,218]. In immature DC, HIV exposure induces membrane extensions through the activation of the Rho-GTPases Cdc42 and promotes viral transfer in CD4+ T-cells [219]. Conversely, *trans*-infection involving LPS-matured DC leads to filopodial extensions of the CD4+ T-cells into the structured pocket of DC [220]. Viral transfer in this context depends on the activation of CD4 molecules at the T-cell surface. DC also express molecules that can attenuate *trans*-infection. Indeed, the expression of proteins involved in control of actin nucleation and stabilization such as TSPAN7 and DNM2 in MDDC, have been demonstrated to display an important role in the limitation of HIV-1 endocytosis and in the maintenance of virus particles on dendrites [221]. Mature DC are more efficient in HIV *trans*-infection compared to immature DC [146]. Of note, a specific DC subset expressing both CD14 and CD16 was demonstrated to have a high capacity to transmit infection to CD4+ T-cells through DC-SIGN expression [222]. Cell-to-cell viral transmission favors HIV dissemination and persistence as it is less sensitive to the presence of antiretroviral drugs such as the nucleotide reverse transcriptase inhibitor tenofovir and the non-nucleoside reverse transcriptase inhibitor efavirenz [223]. Studies in humanized mice also emphasized the importance of DC-mediated *trans*-infection of T-cells in promoting viral persistence [224].

Once the virus interacts with DC, its fate is dependent on the bound receptor, the subset of DC, the stage of maturation and the cell interaction [189]. The majority of virions captured are in part degraded by DC. However, interaction with DC-SIGN does not result in complete viral degradation. Virions bound to DC-SIGN are retained in early endosome compartments and this may promote *trans*-infection [186,196]. Once captured in mature MDDC, HIV is localized within the cholesterol-enriched and tetraspanin-containing compartments where it may be subsequently delivered to target CD4+ T-cells through the exosome pathway [201,225]. Retention of virions in pDC non-acidic early endosomes will activate IRF7, triggering IFN-α production [226]. The pDC up regulate CD83 as well as CCR7 following viral interaction and respond to CCL19-mediated chemotaxis [227]. Nevertheless, HIV infection in pDC does not lead to complete maturation. The pDC stimulated with HIV do not result in high expression of CCR7, CD40 and CD86 compared to pDC activated with TLR7 agonists [226]. In addition, pDC partially matured by HIV induce low levels of CD4+ and CD8+ T-cell proliferation as opposed to pDC stimulated with TLR7 or TLR9 agonists [226]. Furthermore, HIV-exposed pDC produce inferior levels of IL-6 and TNF-α compared to other stimuli. In contrast to ligands that can traffic to late endosome/lysosome, HIV retention in early endosomes leads to poor activation of the NF-κB signaling resulting in incomplete maturation and lack of MHC expression. Therefore, pDC are not able to efficiently present antigens. The induction of the NF-κB signaling pathway leading to complete maturation is closely associated to TLR regulation in which previously activated pDC become resistant to further stimuli that would lead to additional production of IFN-α [189]. Therefore, the lack of maturation in HIV-exposed DC leads to unregulated, persistent production of IFN-α due to repetitive stimulation and this increase in the levels of IFN-α can induce serious immunological consequences including cell exhaustion [189,226]. Other studies reported that the interaction between HIV envelope and CD4 may promote type 1 IFN production by pDC without reaching maturation [228]. Similar to pDC, mDC exposed to HIV exhibit a certain level of activation but do not fully mature [229]. Early studies demonstrated that HIV exposure impairs the immunogenic potential of DC [230]. In contrast to pDC, TLR7-mediated immune response is poorly induced in MDDC following HIV exposition [231]. Partially matured mDC lead to the generation of Tregs, which attenuate the pro-inflammatory antiviral response. Also, alterations of the crosstalk between mDC and NK cells lead to poor DC maturation. Immature MDDC exposed to HIV secrete considerable levels of IL-10 leading to resistance of NK-cell mediated lysis and accumulation of poorly immunogenic DC in lymph nodes of HIV-infected individuals [232]. Innate-like T cells, invariant NKT (iNKT), respond to infected DC as an early immune detection mechanism [233]; however, the presence of Vpu and Nef inhibit mechanism of action performed by the iNKTs.

Evidence provided by various groups including that of Dan Littman support the concept that mDC and MDDC are unable to appropriately sense HIV due to restricted viral replication in these cells; this impedes their maturation process and immunogenic potential [195]. The HIV capsid includes motifs able to block sensing of viral cDNA [234]. Studies by Manel et al. demonstrated that increased HIV replication by ectopic SIV-Vpx expression in human DC results in increased viral replication and immunogenic potential [235]. In contrast, other lines of evidence provided by the groups including that of Vincent Piguet suggest that a decreased immunogenic potential of DC during HIV infection is explained by HIV altering cellular metabolic processes important for antiviral responses [236]. Indeed, autophagy, a process required for TLR-induced immune response, was shown to be exhausted in MDDC during HIV-infection [236]. Interaction with HIV envelope leads to the activation of mTOR in MDDC; this results in suppression of autophagy and impaired TLR signaling. Also, the defect in autophagy upon HIV exposition leads to altered antigen processing and presentation [236].

The fact that DC are poorly infected with HIV compared to CD4+ T-cells is explained by the presence of several host factors shutting down HIV replication. Several restriction factors including TRIM5α, APOBEC3G, BST-2 and SAMHD1 are expressed in DC [189,229,237,238]. The ABOBEC3G is up regulated in immature MDDC following stimulation with type 1 IFN, preventing viral infection [239]. The most effective restriction factor probably remains SAMHD1 [237]. The pDC and mDC were reported to express high levels of SAMHD1 that is not degraded by HIV and this leads to inhibition of viral replication [240]. Before the discovery of SAMHD1, it was well established that SIV-Vpx over expression induces MDDC maturation and production of type 1 interferon [235,241]. The immune activation induced by the presence of Vpx is dependent on interaction of de novo synthesis of viral capsid protein with cellular cyclophilin A leading to subsequent activation of IRF3 [235]. The innate sensor involved in this pathway has been proposed to be cyclic GMP-AMP (cGAMP) synthase (cGAS), a factor sensing reverse-transcribed HIV-DNA [234,242]. Nevertheless, the viral capsid prevents sensing of HIV-DNA by cGAS after reverse transcription [234,243]. The lack of Vpx in HIV-1 may in part explain the inability of mDC to accurately induce priming and expansion of HIV-specific T-cell responses. A recent study on HIV-2 that expresses Vpx, demonstrated that infected individuals show higher mDC activation that may contribute to effective viral control [244]. The effect of Vpx in the activation of mDC appears to be difficult to observe as mDC expressed by 30-fold higher levels of SAMHD1 compared to MDDC [229]. Independently of induction by Vpx, type 1 IFN production itself can inhibit early and late stages of HIV replication [245]. In addition to cGAS, another cell host factor has been reported for the recognition of HIV components. Indeed, Gringhuis et al. just recently identified RNA helicase DDX3 as an HIV-1 sensor that interact with abortive HIV-1 RNA, leading to MDDC maturation and production of type 1 interferon, via the signaling adaptor MAVS [246]. HIV interferes with this mechanism through interaction with DC-SIGN that induce the activation of mitotic kinase PLK1, which inhibit signaling downstream of MAVS. Other host factors may favor HIV replication by annihilating the activation of innate immunity. The host exonuclease TREX1 contributes to viral evasion by degrading reverse transcribed HIV-DNA thus preventing sensing by PRRs; deletion of TREX1 triggers innate immune responses [247]. Therefore, impaired viral immune recognition and blocking of infection at different stages of replication may allow HIV to escape detection by DC.

The impact of HIV on DC is also observed quantitatively. Both pDC and mDC frequency is reduced, starting during early stages of infection and throughout the chronic phase of HIV pathogenesis [248,249]. Nevertheless, it appears that CD163+ DC lacking CD16 expression are increased in frequency during HIV infection compared to CD163− DC expressing CD16 [250]. The frequency of DC is inversely associated with viral load. The decrease in peripheral blood pDC numbers may indicate enhanced recruitment into lymph nodes or other tissues, early death, or a defect in precursor differentiation in the bone marrow [229]. Indeed, during primary SIV infection in rhesus macaques, elevated frequencies of pDC tend to migrate into lymph nodes where they subsequently undergo cell death [251]. The number of peripheral blood pDC is positively correlated to CD4+ T-cell counts [229]. The antiviral therapy only partially restores the number of pDC [249]. During the acute phase of HIV infection, pDC appear to be in a hyperactive mode as they produce superior levels of pro-inflammatory cytokines including IL-6, TNF-α and chemokine ligands of CCR5 following stimulation with TLR-7 agonist [248]. Throughout the acute and chronic phase of infection, pDC constantly persist in being large producers of IFN-α; this is possibly due to impairment in type 1 interferon regulation as these cells partially mature [229]. The HIV does not trigger the signaling pathway involved in shutting down IFN-α expression. In fact, HIV-exposed pDC continuously up regulate IRF7 that is associated with the persistent production of type 1 IFN. High levels of IFN-α in infected primates and humans are correlated with immune activation and disease progression. Persistent production of type 1 IFN is also associated with the T-cell exhaustion reflected by the up regulation of PD-1 and CTLA-4 [252,253,254]. Compared to the non-pathogenic primate model in which the intense expression of IFN-α and IFN-stimulated genes is resolved following primary SIV infection, the pathogenic model of SIV is characterized by sustained levels of type 1 IFN [255,256]. Whether the IFN signature mirrors SIV replication or the control of SIV replication in pathogenic versus non-pathogenic SIV models remains unclear [257,258]. However, several studies report that HIV-exposed pDC produced reduced levels of IFN-α following stimulation by TLR-7 and TLR-9 agonists and display decreased capacity to activate NK cells [259,260,261]. Recent studies demonstrated that Vpu interacts with BST2 and inhibits TLR-7 mediated IFN-α production in pDC [262]. Persistent production of IFN-α can result in enhanced apoptosis of uninfected CD4+ T-cells with the up regulation of the TNF-mediated cell death pathway [263]. Furthermore, sustained type 1 IFN signaling during chronic infection leads to up regulation of PD-1 on DC, production of IL-10 and reduced frequency of CD4+ T-cells producing IFN-γ [264,265]. In addition to HIV, other factors including an altered microbiome appear to be involved in the persistence of immune activation [266]. The high prevalence of Prevotella species in HIV-infected individuals activates mDC which will induce increased T-cell activation.

The effects of type 1 IFN on Th17 polarization are controversial. Although, pDC and IFN trigger exacerbated Th17 responses in a model of psoriasis [267], it was also reported that type 1 IFN decreases the development Th17 responses [268]. The HIV-exposed pDC can induce the generation of Tregs from naive CD4+ T-cells [269]. This induction of Tregs involved in IDO expression is trigged following CD4-mediated endocytosis and TLR7 activation. The generated Tregs by pDC were shown to suppress CD4+ T-cell responses and inhibit maturation of mDC upon stimulation with TLR4 and TLR7 agonist thus reducing antigen-presentation [270]. This finding reflects the dichotomous impact of pDC during HIV infection as previous studies demonstrated that pDC could also induce bystander activation and maturation of mDC through the production of TNFα [227]. Growing evidence indicates that mDC functions are altered within weeks following HIV infection where viremia is still undetectable. During early stages of acute HIV infection, mDC lose the capacity to produce high levels of IL-12 and TNF-α following stimulation with TLR-7 agonist [271]. The MDDC from uninfected individuals exposed to plasma from acutely HIV infected subjects had a reduced ability to secrete pro-inflammatory cytokines. Also, culture with plasma from acutely HIV-infected individuals inhibited NK and T-cell activation induced by MDDC [271]. The HIV itself does not seem to be directly involved in DC suppressed cytokine production. Conversely, non-viral components including apoptotic microparticles seem to affect mDC function, preventing the establishment of much needed innate and adaptive immune response at early stage of infection [271]. As opposed to the early stages of acute HIV infection, mDC from later stages are hyper-responsive to TLR7 agonist and produce strong levels of IL-12, IP-10, TNF-α and ligands of CCR5 [248]. These findings indicate that the alteration in mDC function during acute HIV infection is time specific as it transitions from an absence to a massive production of pro-inflammatory cytokine, which affects the course of immune response. The impairment in mDC functions continues during the chronic phase of HIV infection [229]. The MDDC from chronically HIV-infected individuals produce lower levels of IL-12 upon stimulation with LPS and CD40 ligand [272]. The IL-12 is essential for the activation of NK cells and generation of Th1 cells. Furthermore, MDDC from infected individuals exposed to plasma from untreated viremic subjects have a reduced ability to produce IL-12, IL-6 and TNF-α after stimulation with TLR3 and TLR7 ligands [273]. Again, non-viral components seem to be responsible for the altered functions of MDDC. The direct effect of HIV on MDDC function was shown to involve production of IL-10 that inhibits cell maturation [229].

In addition to their direct role in HIV replication and *trans*-infection, studies by the group of Sharon Lewin revealed the capacity of monocytes and mDC to promote post-integration HIV latency in resting CD4+ T-cells [274].

## 7. Conclusions

Myeloid cells such as monocytes, MΦ and DC represent key components of the innate immune system, being able to detect and signal the presence of pathogens and further instruct the adaptive immune system for efficient control and elimination. Myeloid cells are functionally diverse and comprise well-defined subsets. Novel insights into the myeloid cell diversity are expected now, with the impressive development of transcriptional profiling technologies at single-cell level. In this sense, it is important to emphasize that very recent studies identified classical monocytes as predictors for response to cancer immunotherapy [275]. HIV impairs the functional properties and exploits the biology of myeloid cells to ensure its survival. The recent advances in the ontogeny of myeloid cells, with the discovery of long-lived tissue resident MΦ able to self-renew, raise new questions relative to the role of such myeloid cells during homeostasis and disease [276]. Whether these long-lived myeloid cells contribute to HIV reservoir persistence during ART remains unknown. In the light of the newly proposed trained immunity concept [277], the ability of HIV to act at epigenetic level and imprint myeloid cells and their precursors with specific features, thus shaping the quality of immune response to other pathogens, remains an investigation area of the future. Finally, understanding how changes in the microbiome influence the myeloid cells biology in various tissue swill lead to the discovery of novel therapies.
